# Training the Next Generation of Clinician–Innovators: The Virtual Magic Wand Program

**DOI:** 10.1016/j.xjidi.2022.100108

**Published:** 2022-02-11

**Authors:** Yakir S. Levin, Kachiu C. Lee, Adam B. Raff, Jamie J. Breslin, William Ju, Lilit Garibyan

**Affiliations:** 1 Wellman Center for Photomedicine, Massachusetts General Hospital, Harvard Medical School, Boston, Massachusetts, USA; 2 Department of Dermatology, Massachusetts General Hospital, Harvard Medical School, Boston, Massachusetts, USA; 3 Main Line Center for Laser Surgery, Philadelphia, Pennsylvania, USA; 4 Department of Dermatology, Lewis Katz School of Medicine, Temple University, Philadelphia, Pennsylvania, USA; 5 Department of Dermatology, Beth Israel Deaconess Medical Center, Beth Israel Lahey Health, Harvard Medical School, Boston, Massachusetts, USA; 6 Advancing Innovation in Dermatology, Mendham, New Jersey, USA

**Keywords:** PWS, problem worth solving, VMW, Virtual Magic Wand

To the Editor

A key step in biomedical innovation is problem identification and needs assessment. Clinicians can play an essential role in biomedical innovation because they are at the forefront of patient care and encounter clinical unmet needs on a regular basis (Garibyan and Anderson, 2017; Garibyan et al., 2021; Lee et al., 2021). However, they may lack the skills to deeply define and present the critical elements of the problems they have identified in a concise and focused manner (i.e., the pitch). This important step is necessary before the development of collaborations with research faculty to work together toward solutions.

The Magic Wand Initiative was founded at the Massachusetts General Hospital (Boston, MA) in 2013 to empower clinicians to engage in problem-driven research and innovation. The demonstrated success of the single-institution program (Garibyan and Anderson, 2017; Garibyan et al., 2021) led us to create a pilot training program that could be shared more broadly within dermatology. We developed the Virtual Magic Wand (VMW) program, an interactive course geared toward educating dermatologists to engage in problem-driven innovation.

The VMW program is a 10-month course designed to educate dermatologists to engage in problem-driven innovation by identifying and defining an unmet clinical need in dermatology. The program is advertised by e-mail communication with residency program directors (through the listserv of the Association of Professors in Dermatology) and by announcement at the annual Next Generation of Innovators Luncheon organized by Advancing Innovation in Dermatology. Course meetings are through monthly video conferences, and the curriculum is composed of didactics, interactive group brainstorming, and pitch sessions. In a pitch session, participants present and discuss their chosen clinical problem in a 5-minute presentation. In the presentation, the problem is thoroughly characterized in terms of its clinical importance, its societal and economic impact, and all stakeholders impacted by it. A specification sheet containing the necessary and desirable characteristics of an ideal solution is outlined. The presentation is intended to deeply define the unmet need and motivate listeners, such as research faculty, engineers, and other stakeholders, to join the clinician-led team to work on solutions to the problem. Videoconferences take place on a monthly basis between July and April and usually last for 1‒2 hours (6–8 PM Eastern Time). Before enrolling in the course, each participant who is a resident or fellow is required to provide a letter of support from their program director affirming that they will be granted time to attend the videoconferences. Attendance is monitored at each session, and participants are allowed to miss a maximum of 2 of 10 sessions. Although missing more than two sessions results in removal from the course, to date, this remedy has not been necessary for any participants. The course leadership and invited faculty speakers receive no remuneration for their participation. Advancing Innovation in Dermatology (https://www.advancing-derm.org) provided initial funding for administrative support of the program. No costs are incurred by residency programs whose trainees participate in the program.

The VMW program maintains no ownership of problems identified by course participants, and problems alone are not patentable. Should a scholar or team of scholars opt to pursue their problem worth solving (PWS) by independently developing novel solutions and intellectual property, it will be up to the team members to address the ownership of intellectual property with each other and in accordance with their institutional policies.

Each dermatologist enrolled in the course is asked to identify 2‒3 unmet needs from their clinical practice that they are motivated to characterize. These are then presented in an initial brainstorming session attended by their colleagues and key opinion leaders in the field of dermatology, who assist in vetting and defining the scope of the problems. Participants then choose which problem—their PWS—to further characterize during the course. For PWS pitch sessions, key opinion leaders with specific expertise in the chosen PWS are invited to participate.

Didactic sessions address practical issues to consider when formulating a PWS and its possible solution, such as stakeholders, market research, reimbursement, regulatory issues, and intellectual property concepts. Industry, regulatory, and intellectual property lawyers are also invited to speak about these important steps in the innovation pathway. Depending on the session topic, 2‒4 invited faculty are present for each session. The course culminates in the creation of a white paper dedicated to the characterization of each participant’s PWS and an outline of necessary and desirable characteristics of a solution. A sample course schedule and topic list are depicted in Table 1.

**Table 1:** Sample Course Schedule and Topic

Session Number	Title	Description
1	Introduction to Magic Wand Initiative	Description of the Magic Wand Initiative and its initial implementation at MGH.
2	Brainstorming	Participants present up to three problems that they have identified, with feedback from KOLs and peers.
3	Effective Presentation Skills	How to give a great talk and how to make an effective elevator pitch.
4	Pitching Your Problem I	Participants pitch their PWS; 5-minute talk followed by feedback from KOLs.
5	Lessons Learned	Clinician–innovators share stories and lessons learned on their journey in clinical innovation.
6	Stakeholders	Stakeholders: how you identify them, how you engage them, and what role they play in your program.
7	IP and Regulatory	Important information about IP and regulatory aspects of innovation.
8	Pitching your Problem II	Participants pitch their PWS; 5-minute talk followed by further feedback from KOLs.
9	Beyond Clinical Considerations	Important factors in team formation, company formation, and business model.
10	Final Pitch	Participants present their finalized PWS; White paper due.

Abbreviations: IP, intellectual property; KOL, key opinion leaders; MGH, Massachusetts General Hospital; PWS, problem worth solving.

The VMW program has graduated three classes from 2017 to 2020. There were 7 participating dermatologists in the first class (2017–2018), 9 in the second (2018‒2019), and 17 in the third (2019‒2020). In the third year, the program was modified to enable participants to work in teams; a larger number of participants could therefore be accommodated. Participants were characterized by their demographics, by the PWS they identified, and by their subsequent involvement in dermatologic innovation. In addition, the 17 members of the 2019–2020 VMW class were surveyed before starting and after the completion of the course regarding their interest in and perceived ability to participate in problem-driven innovation.

VMW has graduated 33 dermatologists, of whom 28 were residents, 1 was a clinical fellow, 3 were assistant professors, and 2 were associate professors. A total of 30 (91%) participants were affiliated solely with academic institutions, whereas 1 (3%) was affiliated solely with a private practice. One (3%) participant had affiliations with an academic institution and with a private practice over the course of their VMW participation, and another (3%) had academic and military affiliations. The course has attracted participants from 26 institutions (Table 2). Although women make up only 12% of innovators in the United States, it is notable that 17 (52%) of the VMW participants were female (Nager et al., 2016). Of the 17 female participants, 4 (24%) were faculty (2 assistant professors and 2 associate professors), 1 (6%) was a clinical fellow, and 12 (70%) were residents. Women have achieved gender parity in dermatology training programs, and their representation in VMW may be less surprising. However, it has been shown that female inventors are more likely to innovate in female-focused areas, and their participation in innovation training may result in future efforts focused on problems that would have otherwise been neglected. Specifically, an analysis of drug and medical patents in the PatentsView–National Bureau of Economic Research database defined female-focused patents as those whose text covers female organs, diseases, physiologic processes, and genetics. The authors showed that 14.7% of the patents by female-majority teams were female focused. In contrast, patents by male-majority teams were 12.9% female focused (Koning et al., 2021). For the VMW, it is notable that female scholars chose the PWS related to female pattern hair loss and nipple injury in nursing mothers.

**Table 2:** Affiliations of Course Participants

Brown University
Boston University
Case Western Reserve University
Dermatology Specialists of Spokane
Duke University
Harvard University
Mayo Clinic Rochester
Mount Sinai Medical Center
New York University
Northwestern University
Pilaris Hair Restoration
Rush University
San Antonio Uniformed Health Services Health Education Consortium
Skin Care Physicians
Skin Institute of South Florida
Stanford University
University of California at San Francisco
University of Connecticut
University of Iowa
University of Maryland
University of North Carolina
University of Pittsburgh
University of South Florida
University of Southern California
University of Toronto
University of Utah

Four of the five faculty participants were female. We do not know the reason for the skew toward female participation at the faculty level. Although it could reflect greater appeal to women than to men, it may simply be an artifact of a small sample size or due to the female director (LG) and female cochair (KCL) actively encouraging faculty members to apply. Representation at the leadership level is known to be important in attracting underrepresented members. More investigation and a larger sample size are needed to understand the success of our program in attracting women to innovation

Table 3 lists the PWSs identified and characterized by VMW participants. The PWS was frequently narrowed after input from colleagues and clinician experts.

**Table 3:** Problems Worth Solving Characterized by VMW Participants

**2017‒2018**
Shortcomings in compression stockings
Seborrheic keratosis
Postoperative scarring
Noncompliance with sun protection
Hidradenitis Suppurativa
Drug delivery of topical minoxidil for female pattern hair loss
Prevention of squamous cell carcinoma in organ-transplant recipients
**2018‒2019**
Chronic venous insufficiency
Hand‒foot skin reaction in targeted skin cancer therapy
Dermal hyperpigmentation
Tracking chronic skin diseases
Wound care in inflammatory ulcerating conditions: an education gap in dermatology
Tattoo removal
Physician burnout
Nipple injury and pain in nursing mothers
Difficulty choosing appropriate skincare products
**2019‒2020**
Surgical smoke exposure
Dermatophyte detection
Onychomycosis as a drug delivery problem
Site identification before skin cancer surgery
Protecting skin of color from pigmentation and pigmentary disorders
Alopecia as a problem in need of objective metrics
Diagnostic delay in cutaneous nontuberculous mycobacterial infections
The power cord associated with electrodesiccation devices
Treatment of actinic keratosis in patients at high risk of developing squamous cell carcinoma

Abbreviation: VMW, Virtual Magic Wand.

A total of 29 volunteer faculty have delivered didactic lectures on specific topics or served as key opinion leaders to brainstorm with course participants. The faculty include academic dermatologists (with ranks ranging from assistant to full professor, including three former American Academy of Dermatology presidents), dermatologists in private practice with experience in innovation, and industry representatives with subject matter expertise in specific areas such as intellectual property law and regulatory affairs.

All the 17 participants from the 2019‒2020 class responded to the presurvey, and 11 responded to the postsurvey. Overall, respondents indicated a greater understanding of and ability to engage in key aspects of the process of innovation after completion of the course. For example, 4 of 17 (24%) respondents to the precourse survey agreed or strongly agreed with the statement, “I can identify potential stakeholders/team members to collaborate with when solving problems.” In the post-course survey, 11 of 11 (100%) agreed or strongly agreed with the statement. In the precourse survey, 2 of 17 (12%) respondents agreed or strongly agreed with the statement, “I can manage a team through the innovation process of turning an idea into a product.” In the postcourse survey, 10 of 11 (91%) of respondents agreed or strongly agreed. Table 4 depicts their responses to each of the survey questions. Although the survey shows an obvious positive impact of the course, the limited response (11 responses to the postsurvey and 17 to the presurvey) may positively skew the results. Although it is unclear why six participants did not respond to the survey, we suspect that the conclusion of the program toward the end of the academic year, a time during which residents and fellows are often transitioning to their next professional endeavor, may have resulted in inattention to the e-mail request to complete the survey. To ensure a better response rate to the postsurvey, we plan to require course participants to commit in writing, before their matriculation into the course, to completing the presurvey and postsurveys. In addition, course completion certificates will be provided only after completion of the survey.

**Table 4:** Results of Survey Administered to the 2019 VMW Class Before and after Completion of the Course

Survey Question	Precourse Survey (N = 17)		Postcourse Survey (N = 11)	
	Disagree + Strongly Disagree (%)	Agree + Strongly Agree (%)	Disagree + Strongly Disagree (%)	Agree + Strongly Agree (%)
Express/rate your level of interest in identifying unmet clinical needs and solving them.	0 (0)	17 (100)	0	11 (100)
At work, I can identify and select unmet clinical problems worth solving.	0 (0)	13 (76)	0	11 (100)
I have the time needed to dedicate myself to solving unmet clinical problems.	8 (47)	5 (29)	3 (27)	5 (45)
I can independently generate potential solutions to unmet clinical problems.	4 (24)	5 (29)	1 (9)	10 (91)
I can identify potential stakeholders/team members to collaborate with when solving problems.	6 (35)	4 (24)	0	11 (100)
I can create prototypes to test potential solutions to unmet clinical problems.	10 (59)	3 (18)	0	7 (64)
I can design feasibility (proof-of-concept) studies for potential solutions for unmet clinical problems.	6 (35)	7 (41)	0	8 (73)
I can manage a team through the innovation process of turning an idea into a product.	9 (53)	2 (12)	0	10 (91)
At work, I am involved in biomedical innovation or quality improvement projects.	3 (18)	12 (71)	0	9 (82)
I understand the process of regulatory approval for medical products.	10 (59)	3 (18)	0	8 (73)
I collaborate with intra or inter-department research faculty regularly to solve problems.	8 (47)	4 (24)	2 (18)	8 (73)
I can pitch my idea to investors for my innovative projects.	6 (35)	7 (41)	0	10 (91)
I know what intellectual property (IP) is and its role in biomedical innovation.	6 (35)	5 (29)	0	10 (91)
I know where and how to obtain funding for my innovative ideas.	10 (59)	1 (6)	0	7 (64)

Abbreviation: VMW, Virtual Magic Wand.

Participants were asked to indicate one of five responses to each of the survey questions: strongly disagree, disagree, neutral, agree, and strongly agree. Results are shown in the top two (agree + strongly agree) and bottom two (disagree + strongly disagree) format. Responses that are not depicted in this table were neutral (neither agree nor disagree).

After their introduction to problem-driven innovation through the VMW, 19 (58%) program graduates have become involved in other innovation-based initiatives in dermatology. Three have proceeded to lead other initiatives focusing on problem-driven innovation: one cofounded HealthAI @ Stanford, another cofounded Hacking Dermatology, and a third developed an externship program that pairs early-career physicians with established innovative companies. Hacking Dermatology is an Advancing Innovation in Dermatology program in which teams of participants propose innovative solutions to unsolved problems in Dermatology; winning teams receive seed funding to begin to develop their technologies. Four graduates of the VMW have participated in this externship program. Four graduates have served as analysts on due-diligence teams for an early-stage accelerator fund.

In addition, 11 of the program graduates participated in dermatology-specific hackathons. At the Stanford Health++ 2018 Hackathon, a VMW alumna led SkinSpecs, a team that was awarded Best Understanding of an Unmet Need for its development of a proposal to address the challenge of longitudinal monitoring of chronic skin diseases (Hansen, 2018). A total of 10 graduates have participated in Hacking Dermatology (http://www.hackingdermatology.org). At Hacking Dermatology 2019, VMW participants were represented on three winning teams during the initial round. In the subsequent final pitch competition, teams led by VMW graduates won first, second, and (tied for) third places (Advancing Innovation in Dermatology, 2020).

Nine graduates have cofounded companies. At the beginning of the COVID-19 pandemic, one graduate of our program founded a nonprofit to address the problem of the limited availability of personal protective equipment. The graduate formed a team that developed a mask offering significantly greater protection than conventional surgical masks and which through innovative use of discarded surgical instrument sterilization wrap also has the benefit of supporting environmental sustainability (Brazil, 2020). Another group cofounded a company that has developed a product for pain-free, at-home treatment of warts. The company has won several project competitions and has raised $20.7 million in funding (Hilburn, 2021). This company has also published a patent related to its technology (Waldman et al., 2021). One VMW graduate cofounded a company that has developed technology to enable simple capture and standardization of photos in dermatology clinics, and early access is available (https://www.matchlab.ai/#technology). Table 5 summarizes the contributions to innovation after VMW participation.

**Table 5:** Participation in Innovative Activities after Completion of the VMW Program

Innovative Activity	Number of Graduates
Participated in a dermatology-related hackathon	11
Participated in an externship program pairing early-career participants with innovative companies	4
Cofounded a startup company	9
Served as analysts for an accelerator fund	4

Abbreviation: VMW, Virtual Magic Wand.

In conclusion, the VMW is a pilot program that has shown early success with an impact in teaching problem-driven innovation to dermatologists and engaging them in the process of innovation. The Magic Wand program, which emphasizes the importance of deeply characterizing a clinical problem before countenancing solutions, is applicable to participants at any career level. In the future, we plan to publicize the program more broadly to attract participants at all career stages. Given the success of the program with dermatologists in the United States, our program has already been expanded to Europe. In 2021, we launched the European VMW program to empower, educate, and engage European dermatologists in problem-based innovation (Garibyan, 2021). As these programs mature, objective outcome measures will be needed to quantify the program’s impact. This will include continued surveys of VMW participants before and after participating in the program as well as follow-up surveys and interviews of VMW alumni. Additional longer-term outcome measures will include patent publications; peer-reviewed publications; grant funding for innovative projects; formation of new initiatives and startup companies; as well as funding of such initiatives by nonprofit organizations or venture capital, product approvals, and impact on patient care. We also plan to improve the program by obtaining funding to create a standardized and scalable multimedia education curriculum that can be accessed by any dermatologist interested in learning the process of biomedical innovation.

Problem identification and characterization by clinicians is an essential first step in problem-driven innovation. After identification and characterization of a problem, a collaborative research team can be formed —or joined—to seek a solution. The VMW shows promise as an educational venue to encourage problem-driven innovation in dermatology and serves as an opportunity to seed collaboration between clinical and research faculty.

### Contact information

Further information about the Magic Wand Initiative can be found at magicwandinitiative.org. For further information, please contact info@magicwandinitiative.org. Hacking Dermatology is an initiative of Advancing Innovation in Dermatology. Further information can be found hackingdermatology.org.

### Data availability statement

No datasets were generated or analyzed during this study, except for the survey response data, which are presented in the manuscript in their entirety.

## ORCIDs

Yakir S. Levin: http://orcid.org/0000-0001-8998-0030

Kachiu C. Lee: http://orcid.org/0000-0003-2107-8985

Adam B. Raff: http://orcid.org/0000-0002-3389-5096

Jamie J. Breslin: http://orcid.org/0000-0001-7868-5669

William Ju: http://orcid.org/0000-0002-2193-8917

Lilit Garibyan: http://orcid.org/0000-0002-9266-0887

## Conflict of Interest

The trademark for Magic Wand Initiative is owned by the Massachusetts General Hospital (Boston, MA), which is the current employer of YSL and LG. The authors state no other conflict of interest. Some initial funding for this program was provided by Advancing Innovation in Dermatology, which is a nonprofit organization where JJB and WJ currently work.

## References

[bib1] (2020). Advancing Innovation in Dermatology. Hacking dermatology 2019 program.

[bib2] Brazil B. (2020). https://www.latimes.com/socal/daily-pilot/entertainment/story/2020-04-29/uc-irvine-and-local-business-team-up-to-produce-new-type-of-mask.

[bib3] Garibyan L. (2021). https://www.imcas.com/en/academy/blog/677/interview-with-dr-lilit-garibyan.

[bib4] Garibyan L., Anderson R.R. (2017). Increasing clinical faculty engagement in problem-driven research: the “Magic Wand” initiative at Massachusetts General Hospital. JAMA Dermatol.

[bib5] Garibyan L., Kroshinsky D., Freeman E., Sakamoto F.H., Anderson R.R. (2021). A strategy for empowering clinicians and increasing innovation: the Magic Wand Initiative. Arch Dermatol Res.

[bib6] Hansen A.J. (2018). https://scopeblog.stanford.edu/2018/11/16/hackathon-prize-winner-seeks-to-remotely-monitor-patient-skin-conditions/.

[bib7] Hilburn J. (2021). https://www.forbes.com/sites/jairhilburn/2021/09/22/this-startup-raised-207-million-to-develop-a-kid-friendly-wart-treatment/?sh=7c53e6a860d0.

[bib8] Koning R., Samila S., Ferguson J.P. (2021). Who do we invent for? Patents by women focus more on women’s health, but few women get to invent. Science.

[bib9] Lee K.C., Lee I., Okhovat J.P., Ko J., Powers J.G., Ellis D.L. (2021). Innovation interest within dermatology: a needs assessment for novel thought processes. Arch Dermatol Res.

[bib10] Nager A., Hart D.M., Ezell S.J., Atkinson R.D. (2016). https://itif.org/publications/2016/02/24/demographics-innovation-united-states.

[bib11] Waldman R., DeWane M., Lee M.H., Durso L.S. (2021). https://patents.google.com/patent/US20210154456A1/en.

